# Editorial: Deep brain stimulation for neuropsychiatric disorders: Current status and perspectives

**DOI:** 10.3389/fneur.2022.1029102

**Published:** 2022-11-10

**Authors:** Eduardo Joaquim Lopes Alho, Juan Carlos Baldermann, Luis Eduardo Coutinho Castelo-Branco, William Omar Contreras Lopez

**Affiliations:** ^1^Clinica de Dor e Funcional-CDF, São Paulo, Brazil; ^2^Department of Psychiatry and Psychotherapy, University of Cologne, Cologne, Germany; ^3^Spaulding Rehabilitation Hospital, Boston, MA, United States; ^4^International Neuromodulation Center-NEMOD, Floridablanca, Colombia

**Keywords:** deep brain stimulation, obsessive compulsive disorder, major depressive disorder, drug addiction, aggressive behavior, Tourette syndrome

For over two decades, the value of deep brain stimulation (DBS) in the treatment of different neuropsychiatric diseases has been studied. Over the years, the field has made substantial progress and a number of effective or promising clinical applications have been introduced, e.g., for treatment-refractory obsessive-compulsive disorder (OCD, Tourette Syndrome, major depressive disorder (MDD) and others. Despite this effort, DBS must still be considered a niche topic for both clinicians and the broader field of neuroscience, even in indications with convincing evidence for effectiveness (https://pubmed.ncbi.nlm.nih.gov/35840727/). In this special issue, we aimed to expand the horizon of the interested reader and give a stage to recent advances in DBS for neuropsychiatric disorders.

This Research Topic consists of 8 articles reviewing the current status and perspectives of Deep Brain Stimulation (DBS) for Neuropsychiatric Disorders. Fifty authors from 6 different countries contributed to this topic, leading to almost 15,000 online views and circa 3,000 article downloads at the time of this writing. The majority of the papers were Reviews, but also Opinion, Perspective and Original Research papers were published. The topic theme “DBS for Neuropsychiatric Disorders” was deliberately broadly formulated to cover different diseases and different anatomical targets. Although much effort has been done in recent years regarding the whole of DBS for neuropsychiatric indications, data are still sparse and derived from small open-label studies and therefore this collection can help the reader to have a broader impression of the current status of the DBS therapy also for non-stablished indications. The articles published in this Research Topic covered the treatment of aggression and self-destructive behavior, obsessive-compulsive disorder (OCD), resistant depression (MDD), Tourette syndrome (TS) and drug addiction with DBS.

The paper *Treating Agression and Self-destructive Behaviors by Stimulating the Nucleus Accumbens: A Case Series* (Harat et al.), reports the results of nucleus accumbens stimulation in 6 patients with self-destructive aggressive behavior, from various etiologies. The authors report substantial reduction in self-destructive and aggressive behavior (by 30–100% and by an average of 74.5%, more pronounced in patients with Tourette syndrome. Although other targets have been used in the last years, such as the posteromedial hypothalamus (1) and amygdala (2), the nucleus accumbens has both anatomical and neurophysiological foundations to be elected as a target for the control of aggression, and this case series is one of the first reports for this indication. These results introduce the ventral striatum as an alternative target for such patients, who are often very challenging to manage.

The systematic review *Target-Specific Effects of DBS for Tourette Syndrome: A Systematic Review and Meta-Analysis* (Wehmeyer et al.) evaluated whether DBS is effective in reducing TS symptoms and target-specific differences. In this comprehensive review (65 studies and 376 patients included), the four most commonly used targets for Tourette syndrome (TS) were studied (centromedian nucleus of thalamus with ventrooralis internus and parafascicularis, posteroventrolateral GPi and anteromedial GPi). When the GPi was targeted, there were increased score reductions compared to the thalamic targets, but the authors conclude that DBS is a clinically effective treatment option for treatment-refractory TS. Some questions remain unanswered such as: is there a best target for TS? Are there specific clinical features that would improve in different targets? If so, what are the best candidates for each one (3)?

Regarding *Neuromodulation of Obsessive-Compulsive Disorder (OCD): A review of invasive and non-invasive methods* (Kammen et al.), discussed the rationale and theory behind neuromodulation in OCD. The authors provided an extensive overview of the cortico-striato-thalamo cortical loops involved in OCD, pointing the role of the latero orbitofrontal-ventromedial caudate pathway, limbic network, default mode network, and salience network in the development of the symptoms. This theoretical basis was then translated to provide anatomical and physiological basis for Transcranial Magnetic stimulation (TMS), Transcranial direct and alternate current stimulation (tDCS, tACS), vagus nerve stimulation (VNS) and DBS of various targets. An important message of this interesting review is that mechanistic studies aiming to understand the functional networks implicated in OCD are key to identifying optimal targets. Planning DBS based in connectomic analysis might improve our results in the next years not only for motor diseases, but also for complex psychiatric conditions, such as OCD (4) and depression (5).

In line with this concept, the paper *White Matter Tracts Associated with Deep Brain Stimulation Targets in Major Depressive Disorder: A Systematic Review* (Yu et al.), reviewed and summarized current targets for Major Depressive Disorder (MDD) and relevant diffusion tensor imaging (DTI) studies. Ninety-five studies were included, showing 7 different DBS targets for MDD and 9 white matter tracts with microstructural abnormalities reported. The authors show that each DBS target corresponds to dysfunctional brain regions and circuits associated with different MDD subtypes. They present a taxonomy of DBS targets based on their associated white matter tract and modulated dysfunctional brain circuits and propose DTI-based personalized targeting strategy. Also it would be very interesting to further explore this idea, trying to correlate such imaging subtypes with clinical features. It is possible that, within the wide range of MDD symptoms, some are more or less correlated with the microstructural changes in white matter and would respond better to a chosen target stimulation.

Exploring the subcallosal cingulate cortex (SCC) target for MDD, Sobstyl et al. included in this collection a systematic review entitled *Subcallosal Cingulate Cortex Deep Brain Stimulation for Treatment-Resistant Depression: A Systematic Review. Fourteen* studies were identified with a total of 230 patients with treatment-resistant depression who underwent SCC DBS. The results showed that DBS response and remission rates are not significantly higher than the rate of non-responders and patients without remission. As the authors state, these conclusions should be viewed with caution, since it could be due to small sample size and lack of control groups. The ideas for improving the accuracy and results are in line with the idea of personalizing targets with neuroimaging and individual features.

Marquez-Franco et al. also analyzed the characteristics of the cortico-subcortical connections to explain DBS results in OCD and MDD. The study *Deep Brain Stimulation Neuromodulation for the Treatment of Mood Disorders: Obsessive Compulsive Disorder and Treatment Resistant Depression* reviewed the anatomical background, neurochemical substrates of MDD and OCD, imaging studies and DBS target size, location and stimulation parameters for those conditions.

Drug addiction treatment is also an interesting and important topic, addressed by the paper *Deep Brain Stimulation in Drug Addiction Treatment: Research Progress and Perspective* (Chang et al.). The authors review the neurobiology of drug addiction, and the postulated mechanism of action of DBS in such conditions. The nucleus accumbens was considered the most promising target for drug abuse, but positive effects were also described in other brain regions. Different substances were subject of study (tobacco, alcohol, cocaine, opioids and methamphetamine/amphetamine), but the data is still preliminary, due to lack of double-blinded studies and clinical trials in larger cohorts.

Finally, one might ask, after over two decades of DBS for psychiatric indications, *Why Has Deep Brain Stimulation Had So Little Impact in Psychiatry?* Mocking et al. debate in this interesting paper, that it is not the lack of patients, knowledge, technology or efficacy of DBS that hinders its development and applications in psychiatry. The reasons behind this issue are discussed in the article, and for the sake of the thrill, no spoilers should now be disclosed! What we, however, can presently disclose, is that the motivation for this Research Topic was the will and the need to change this scenario for the years to come.

## Author contributions

All authors listed have made a substantial, direct, and intellectual contribution to the work and approved it for publication.

## Conflict of interest

The authors declare that the research was conducted in the absence of any commercial or financial relationships that could be construed as a potential conflict of interest.

## Publisher's note

All claims expressed in this article are solely those of the authors and do not necessarily represent those of their affiliated organizations, or those of the publisher, the editors and the reviewers. Any product that may be evaluated in this article, or claim that may be made by its manufacturer, is not guaranteed or endorsed by the publisher.
